# The Appropriateness of Glycerin Enema in Pediatric Patients Visiting the Emergency Department

**DOI:** 10.3390/children8050364

**Published:** 2021-05-02

**Authors:** Min-Jung Kim, Yoo-Jin Choi, Jin-Hee Lee, Hyuksool Kwon, Dongbum Suh

**Affiliations:** 1 Department of Emergency Medicine, CHA Bundang Medical Center, CHA University, Gyeonggi-do 13496, Korea; mjtear@naver.com; 2 Department of Emergency Medicine, Ajou University School of Medicine, Gyeonggi 16499, Korea; choiyj0729@naver.com; 3 Department of Emergency Medicine, Seoul National University Bundang Hospital, Seoul National University College of Medicine, Gyeonggi 13620, Korea; jinuking3g@naver.com (H.K.); dongbumes@naver.com (D.S.)

**Keywords:** pediatric, fecal impaction, glycerin enema

## Abstract

Objectives: We determined whether glycerin enemas were appropriately prescribed in pediatric fecal impaction patients using the Leech score and identified factors that influenced the prescription of glycerin enemas in the pediatric emergency department (PED). Methods: We included patients who received a glycerin enema at the PED of a tertiary teaching hospital. We divided the study subjects into two groups on the basis of their Leech scores: an appropriate enema group (Leech score ≥ 8), and an inappropriate enema group (Leech score < 8). Logistic regression was performed to determine the factors associated with glycerin enema administration. Results: The data of 998 patients, including 446 patients in the inappropriate enema group (Leech score 5.2 ± 1.7) and 552 patients in the appropriate enema group (Leech score 10.1 ± 1.7), were analyzed. A discharge diagnosis of fecal impaction was observed significantly more frequently (57.1%) in the appropriate enema group, and nonspecific abdominal pain (8.3%) and acute gastroenteritis (40.8%) were diagnosed significantly more frequently in the inappropriate enema group (*p* < 0.05). Constipation (2.8%) and irritability (3.0%) were slightly more common in the appropriate enema group than in the inappropriate enema group (*p* < 0.05). According to multiple logistic regression, subjects aged 2–8 years (2–4 years, OR 4.24; 4–8 years, OR 2.83), with vomiting (OR 1.72), with irritability (OR 4.52), and with a prolonged last defecation day (OR 1.2) were most likely to receive appropriate enema administration (*p* < 0.05). Conclusion: The results showed that in those aged 2–8 years, with vomiting and irritability, and with a prolonged last defecation day, an enema was generally administered appropriately.

## 1. Introduction

Abdominal pain is a common cause of pediatric emergency department (PED) visits in children [1,2,3,4,5]. In one-third of patients with abdominal pain, the pain is caused by constipation [3,4], and 81% of patients with constipation experience abdominal pain.

The reason why most pediatric patients with constipation visit the emergency department is because of secondary symptoms such as fecal impaction, and in most cases, disimpaction is necessary [6].

Enema administration is a procedure that can cause discomfort in a child [4,6]. It is also known to cause various complications, such as colon damage due to physical stimulation by an enema catheter, ischemic colitis, and malignant hyperthermia after undergoing a glycerin enema [7,8,9,10]. Therefore, it is important to prescribe enema appropriately for relevant situations.

There are some studies on the efficacy and safety of glycerin enemas [4,6,11,12,13], however, there has been no study about the appropriate prescription of glycerin enemas in crowded emergency departments.

The aim of this study was to determine whether glycerin enemas were appropriately prescribed for pediatric fecal impaction patients using the Leech score and to identify factors that influence the prescription of glycerin enema.

## 2. Materials and Methods

### 2.1. Study Design and Setting

We retrospectively reviewed the electronic medical records of patients under the age of 15 who visited the PED from 1 January 2017 to 31 December 2017 at a tertiary teaching hospital that sees 20,000 pediatric patients annually. Pediatric patients are treated by emergency physicians or pediatric physicians supervised by board-certified pediatric emergency attending physicians. This study was approved by the hospital’s institutional review board.

### 2.2. Selection of Participants

We included patients who underwent a glycerin enema while in the PED. Patients were excluded from the study if they had previous history of other natural causes, such as anal stenosis, congenital megacolon, imperforated anus, or other systemic diseases.

### 2.3. Data Collection

The medical records of the patients were reviewed, and data were collected using a standard patient record form.

We collected variables such as age, sex, weight, vital signs (blood pressure, pulse rate, respiratory rate, and body temperature), chief complaint and accompanying symptoms, location of abdominal pain, underlying diseases, previous medications (antibiotics, probiotics, laxatives, etc.), constipation history, abdominal tender points, laboratory results (white blood cells (WBCs), C-reactive protein (CRP), total carbon dioxide (TCO_2_), venous blood gas analysis (VBGA), pH, base excess (BE), and bicarbonate (HCO_3_)), abdominal X-ray findings, the number of glycerin enema attempts, intravenous or oral hydration solution, medications in the emergency department (ED) or discharge prescriptions, grade of the treating physician prescribing glycerin enema, diagnosis at discharge, length of stay, etc.

### 2.4. Procedures

We divided the study subjects into two groups using the Leech score: an appropriate enema group and an inappropriate enema group.

The Leech score was divided into three parts based on the total colon: the right, left, and rectosigmoid colon. Each colon section was assigned a score from 0 to 5. A score of 0 = no feces, 1 = a scant amount of feces, 2 = a small amount of feces, 3 = a moderate amount of feces, 4 = a large amount of feces, and 5 = a large amount of feces with bowel dilatation. The total score ranged from 0 to 15 points. A total score of 8 or more was considered indicative of a diagnosis of fecal impaction [14,15,16].

The same abdominal radiographs were scored by two pediatric emergency physicians. Reliability was assessed by the intraclass correlation coefficient (ICC) of continuous data from different observers. The number of cases required to perform the necessary analysis was determined as in Bonett et al. [17].

The required sample size was calculated; 78 patients were required to produce a two-sided 95% confidence interval with a width of 0.200 when the estimated intragroup correlation for each of the two measurements was 0.750. Data were analyzed using a two-way random-effects ANOVA model.

### 2.5. Outcomes

The primary outcome was to identify factors predictive of appropriate enema administration.

### 2.6. Statistical Analyses

Continuous variables were analyzed using Student’s *t*-test, and if the data were not normally distributed, the Wilcoxon rank-sum test was used as a nonparametric method. Categorical variables were analyzed by Fisher’s exact test, and the results are presented as ratios. A *p*-value less than 0.05 was considered statistically significant. Logistic regression was performed to determine the factors associated with glycerin enema implementation. All statistical analyses were performed using STATA.

## 3. Results

In total, 1076 patients under the age of 15 visited the PED and received a glycerin enema from 1 January 2017 to 31 December 2017.

A total of 78 patients were excluded: 5 patients had underlying systemic diseases, 61 patients did not undergo abdominal X-ray examination, 10 patients did not proceed with the enema procedure, and 2 patients did not have evaluable abdominal X-ray images (no images of the rectosigmoid segment).

**Figure 1:**
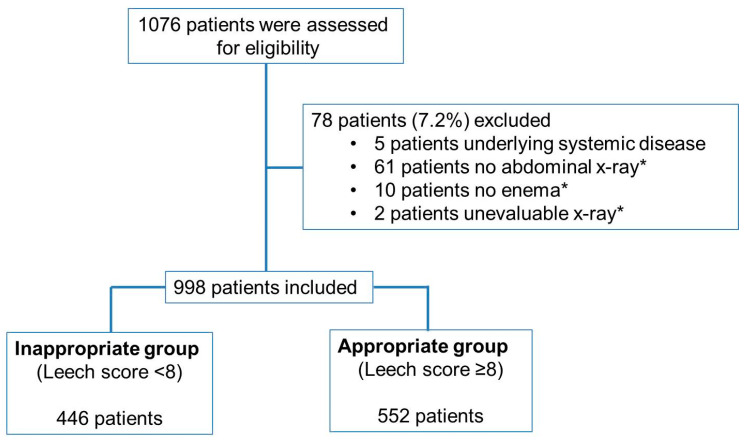
Patient flow chart after application of the inclusion and exclusion criteria. The study selection process after applying the inclusion and exclusion criteria is shown. * no abdominal X-ray: did not undergo abdominal X-ray. * no enema: did not proceed with the enema procedure. * un-evaluable X-ray: no images of the rectosigmoid segment.

A total of 998 patients were finally included. Among them, 446 patients were included in the inappropriate enema group (Leech score < 8) and 552 patients were included in the appropriate enema group (Leech score ≥ 8) (Figure 1).

We divided the study subjects into two groups based on the Leech cutoff value of 8 points; those with a Leech score less than 8 points were assigned to the inappropriate enema group, and those with more than 8 points were assigned to the appropriate enema group. The mean Leech score of researcher 1 was 10 (±3.11) and that of researcher 2 was 8.2 (±2.9). The ICC was acceptable, at 0.884, and was statistically significant (95% CI 0.82–0.93, *p* < 0.05).

A total of 446 patients in the inappropriate enema group and 552 patients in the appropriate enema group were analyzed. The Leech scores were 5.2 (±1.7) in the inappropriate enema group and 10.1 (±1.7) in the appropriate enema group.

The variables of sex, hospital admission from the emergency department (ED), treating physician grade, visit to an outpatient department due to the same cause, emergency department readmission, constipation history, laboratory test results, ultrasound results, number of enemas, and intravenous hydration before glycerin enema were not significantly different between the groups (Table 1).

The most common discharge diagnoses were fecal impaction in the appropriate enema group and nonspecific abdominal pain and acute gastroenteritis in the inappropriate enema group; the differences were significant. More patients in the inappropriate enema group than in the appropriate enema group visited outpatient clinics. The unplanned outpatient number was slightly higher in the inappropriate enema group than in the appropriate enema group (Table 1).

Gastrointestinal symptoms such as constipation and irritability were slightly more common in the appropriate enema group than in the inappropriate enema group. Intravenous hydration after the enema was more common in the inappropriate enema group than in the appropriate enema group. Oral hydration therapy was also prescribed more often in the inappropriate enema group (Table 1).

Multiple logistic regression revealed that subjects aged 2–8 years, with vomiting, with irritability, and with a prolonged last defecation day generally received appropriate enema administration.

On the other hand, the sex, treating physician grade, whether laboratory or ultrasound tests were done, and history of constipation did not affect the appropriate administration of enema in the two groups (Table 2).

## 4. Discussion

To our knowledge, this is the first study to determine whether enemas were administered appropriately in patients in a busy PED and identify factors associated with appropriate enema administration. Our data showed that appropriate glycerin enema administration was not associated with the physician grade, history of constipation, etc. It was significantly associated with age (2–8 years) and symptoms of vomiting, irritability, and prolonged last defecation day.

Abdominal pain is one of the most frequent complaints of pediatric patients visiting the ED [1,2,3,4,5]. Shakya KN et al. [18] reported that approximately 10% of patients with abdominal pain presented to the PED. Twenty-eight percent of pediatric patients with abdominal pain had constipation [3]. A prospective study from a single emergency department in Korea found that fecal impaction and constipation accounted for 21% of gastrointestinal disorders [19,20].

The definition of pediatric constipation according to the North American Society for Pediatric Gastroenterology and Nutrition (NASPGN) is delayed defecation or difficulty defecating more than twice a week, causing discomfort to the patient [1,3,11,21,22,23].

Diagnosing constipation can be done not only clinically, but also using various tools such as abdominal anal examination, clinical physiatric assessment, and instrumental evaluation. It is a disease that is easily encountered but has a variety of diagnostic approaches [24,25].

Fecal impaction refers to hard, large stool masses that are retained in the intestine [26,27]. Fecal impaction is diagnosed by abdominal palpation, rectal examination, and X-ray examination [27,28]. Abdominal palpation and rectal examination may be limited depending on the patient’s cooperation and previous laxative consumption [29]. The NASPGN guidelines recommend X-ray examination in patients with suspected fecal impaction who reject a digital rectal exam (DRE) [5,16,29].

The Barr, Blethyn, and Leech scoring systems are methods for diagnosing fecal impaction on X-ray images. The Barr score was proposed by Barr et al. in 1979; it evaluates the quality and quantity of feces in 4 bowel segments on abdominal X-rays (ascending, transverse, descending colon, and rectum) [16,29]. The sensitivity and specificity of the Barr score were 80% and 90%, respectively [29,30]. The Blethyn score is a simplified scoring system proposed by Blethyn et al. in 1995. It is a method of classifying fecal loading into 4 different grades. The sensitivity and specificity of the Blethyn score were 79% and 92%, respectively [29]. The Leech score is a scoring system proposed by Leech et al. in 1999, which evaluates the feces amount in 3 bowel segments (right colon, left colon, and rectosigmoid colon). The sensitivity and specificity of the Leech score were 76% and 75%, respectively [15,29,30]. Among these systems, there is no single recommended scoring system [16]. In research studies, the Blethyn score is known to have poor interobserver agreement, while the Barr and Leech scores have good interobserver agreement [14,16]. The Leech score has been studied the most, and in recent studies, the diagnostic accuracy of the Leech score was 90% higher than those of the Barr and Blethyn scores [5,15,29].

Most pediatric patients with constipation visit the emergency department because of secondary symptoms such as fecal impaction, and in most cases, disimpaction is necessary [6,31].

Treatment of fecal impaction includes enema administration, oral laxatives, and stool removal under general anesthesia, but none have been determined as the optimal treatment [4,6,12]. Enemas are a noninvasive, simple method of disimpaction and effective for immediate symptom relief [6]. However, pediatric patients dislike enemas due to discomfort [27], and enemas can cause various complications [7,8,9,10]. Therefore, enemas should be administered in only situations in which it is necessary.

According to our data, among the total enema patients, 55% had fecal impaction based on the Leech score (≥8), and an enema was administered in 25% of non-fecal impaction patients (Leech score < 8). These results suggest that unnecessary enema administration is often performed; thus, it seems necessary to educate and improve the knowledge of medical staff.

We expected that the experience of the physician and a past history of constipation would reduce unnecessary enema administration, but our data showed that these variables did not have an effect.

The large number of patients aged 2–8 in the appropriate enema group was likely due to the high incidence of fecal impaction in this age group and the age at which children can communicate. Additionally, the more gastrointestinal symptoms, such as constipation and irritability, the child had, the more often an enema was administered appropriately. 

In contrast, abdominal pain and vomiting were more common in the inappropriate enema group than in the appropriate enema group. Stephen B. Freedman et al. showed that patients with fecal impaction who visited the emergency department mainly complained of gastrointestinal symptoms (abdominal pain and vomiting) [6]. Unlike previous studies, the fecal impaction group, determined by the Leech score, had more symptoms, such as constipation and irritability, than the non-impaction group. 

Apart from symptoms, nonspecific abdominal pain and acute gastroenteritis were more common diagnoses in inappropriate enemas. Nonspecific abdominal pain is a diagnosis that leads to abdominal pain that is less likely to be an organic cause [32]. Acute gastroenteritis refers to symptoms of diarrhea or vomiting [33]. It is interpreted that, regardless of diagnosis, enemas were often performed to improve symptoms (abdominal pain, vomiting, etc.). Enemas were a commonly used treatment for children who visited the emergency room for gastrointestinal symptoms. However, the decision-making for the enema depended on the doctor’s experience and hospital policy, rather than the exact diagnostic decision-making. Therefore, it is necessary to diagnose fecal impaction and conduct disimpaction through appropriate diagnostic pathways.

There were some limitations to our study.

First, this was a retrospective study. Therefore, it is possible that selection bias and information bias occurred during the data extraction process. However, to minimize the possibility, two physicians independently extracted data, and if there was a discrepancy between the two, it was discussed until an agreement was reached.

Second, it was difficult to judge the appropriateness of enema administration because it was not classified according to the definition of fecal impaction but was classified according to the Leech score. However, due to the limitations of this retrospective study, this was inevitable. 

Third, there will be cultural differences in implementing Enema. Therefore, it is difficult to generalize as a result of this study.

Finally, in this study, we failed to find any factors affecting the inadequate enema. However, the study was a retrospective study, and there were limitations that were evaluated using one diagnostic tool using Leech score. In the future, a prospective registry and study is needed to apply an accurate diagnosis and treatment pathway using the diagnostic criteria of fecal impaction.

## 5. Conclusions

We analyzed the appropriateness and the factors affecting proper enema administration in patients undergoing glycerin enema. It was found that in those aged 2–8 years old, with vomiting and irritability, and with a prolonged last defecation day, enemas were generally administered appropriately.

## Figures and Tables

**Table 1:** Participants’ characteristics.

	Leech Score		*p*-Value
	<8 Inappropriate (N = 446)	≥8 Appropriate (N = 552)	
Age (year), mean ± SD	4.2 ± 3.1	3.6 ± 2.4	0.000
Male, N (%)	252 (56.5)	299 (54.2)	0.461
Admission, N (%)	14 (3.1)	17 (3.1)	0.957
Diagnosis, N (%)			0.002
Fecal impaction	193 (43.3)	315 (57.1)	
Nonspecific abdominal pain	37 (8.3)	29 (5.3)	
Acute gastroenteritis	182 (40.8)	161 (29.1)	
Acute gastritis	2 (0.5)	0 (0)	
Acute appendicitis	3 (0.7)	2 (0.4)	
Intussusception	1 (0.2)	0 (0)	
Upper respiratory infection	21 (4.7)	35 (6.3)	
Lower respiratory infection	4 (0.9)	4 (0.7)	
Urinary tract infection	2 (0.5)	1 (0.2)	
Physician grade, N (%)			0.900
Low grade resident	344 (77.5)	437 (79.3)	
High grade resident	13 (2.9)	16 (2.9)	
Pediatric emergency specialist	61 (13.7)	67 (12.2)	
Revisit, N (%)			
OPD_appointment	140 (32.9)	138 (25.5)	0.029
OPD_visit	99 (22.6)	90 (16.6)	0.017
OPD_Same cause	51 (15.7)	46 (14.9)	0.529
ED Revisit	27 (6.2)	33 (6.1)	0.955
Gastrointestinal symptoms, N (%)			0.035
Abdominal pain	290 (65.9)	335 (63.3)	
Vomiting	140 (31.8)	156 (29.5)	
Constipation	5 (1.1)	15 (2.8)	
Irritability	4 (0.9)	16 (3.0)	
Flank pain	1 (0.2)	2 (0.4)	
Poor oral intake	0	4 (0.8)	
Melena	0	1 (0.2)	
Previous constipation history, N (%)	82 (23.9)	102 (27.8)	0.238
Laboratory test, N (%)	133 (29.8)	142 (25.7)	0.150
Ultrasound, N (%)	38 (8.5)	50 (9.1)	0.766
The number of enemas (≥2), N (%)	8 (1.8)	11 (2.0)	0.821
Oral hydration therapy, N (%)	72 (16.1)	57 (10.3)	0.006
IV hydration before enema, N (%)	87 (19.5)	112 (20.3)	0.758
IV hydration after enema, N (%)	64 (14.4)	53 (9.6)	0.02
Length of stay in ED, min, mean ± SD	181.3 ± 146.0	176.6 ± 164.0	

OPD: Out-patient department. ED: emergency department. IV: intravenous.

**Table 2:** Variables associated with the appropriate decision to do a glycerin enema.

	Univariate Analysis				Multivariate Analysis			
	OR	Standard Error	*p*-Value	95% CI	OR	Standard Error	*p*-Value	95% CI
Gender								
Male	1							
Female	1.10	0.14	0.461	0.86–1.41	1.07	0.19	0.687	0.76–1.52
Age (years)	0.93	0.02	0.001	0.89–0.97				
<1	1							
1–2	1.66	0.53	0.114	0.89–3.11	2.09	1.06	0.147	0.77–5.67
2–4	2.44	0.74	0.003	1.35-4.41	4.24	2.11	0.004	1.59–11.26
4–8	1.69	0.50	0.079	0.94–3.03	2.83	1.43	0.039	1.05–7.61
8–14	1.15	0.40	0.689	0.58–2.26	2.44	1.35	0.108	0.82–7.23
≥14	0.23	0.26	0.194	0.03–2.09	-			
Physician grade								
Low grade resident	1							
High grade resident	0.97	0.37	0.934	0.46–2.04	0.97	0.54	0.960	0.33–2.91
Pediatric emergency specialist	0.86	0.17	0.446	0.60–1.26	0.82	0.22	0.459	0.48–1.89
Gastrointestinal symptoms								
Abdominal pain	1							
Vomiting	0.96	0.14	0.799	0.73–1.27	1.72	0.41	0.023	1.08–2.76
Constipation	2.6	1.36	0.068	0.93–7.23	2.41	1.55	0.170	0.69–8.48
Irritability	3.46	1.96	0.028	1.15–10.47	5.32	4.52	0.049	1.00–28.15
Flank pain	1.73	2.12	0.655	0.16–19.19	-			
Constipation history	1.23	0.21	0.238	0.87–1.72	0.97	0.21	0.906	0.64–1.49
Last defecation day	1.22	0.76	0.002	1.08–1.38	1.2	0.10	0.034	1.01–1.42
Laboratory test	0.82	0.12	0.150	0.62–1.08	1.14	0.46	0.738	0.52–2.51
Ultrasonography	1.07	0.24	0.766	0.69–1.66	1.43	0.51	0.314	0.71–2.87
IV hydration pre enema	1.05	0.17	0.758	0.77–1.44	1.3	0.48	0.477	0.63–2.67
IV hydration post enema	0.63	0.13	0.021	0.43–0.93	0.86	0.32	0.679	0.42–1.75

IV: Intravenous.

## Data Availability

https://www.dropbox.com/s/0fi8ggv68hln0sl/enema_CRF_ver%20%28%EA%B3%B5%EA%B0%9C%29.xlsx?dl=0 (accessed on 2 May 2021).
